# The Use of Digital Health in the Detection and Management of COVID-19

**DOI:** 10.3390/ijerph17082906

**Published:** 2020-04-23

**Authors:** Meshari F. Alwashmi

**Affiliations:** 1 School of Pharmacy, Memorial University of Newfoundland, Health Sciences Centre, 300 Prince Philip Drive, St John’s, NL A1B 3V6, Canada; mfa720@mun.ca; 2 Chief Scientific Officer, BreatheSuite Inc., St John’s, NL A1B 2X2, Canada

**Keywords:** digital health, mHealth, infectious disease, COVID-19

## Abstract

Digital health is uniquely positioned to enhance the way we detect and manage infectious diseases. This commentary explores the potential of implementing digital technologies that can be used at different stages of the COVID-19 outbreak, including data-driven disease surveillance, screening, triage, diagnosis, and monitoring. Methods that could potentially reduce the exposure of healthcare providers to the virus are also discussed.

## 1. Introduction

In December of 2019, hospitals began to report cases of unidentified pneumonia among patients with a history of exposure to the Huanan seafood market in Wuhan, Hubei, China. Researchers rapidly isolated a novel coronavirus (SARS-CoV-2, also referred to as COVID-19) from confirmed infected pneumonia patients [1]. Attracting great attention nationally and worldwide, confirmed cases of COVID-19 exceeded those of severe acute respiratory syndrome (SARS) and Middle East respiratory syndrome (MERS). As of 13 April 2020, confirmed cases of COVID-19 had exceeded 1,800,000 cases and 117,000 fatalities. The World Health Organization (WHO) has recently declared COVID-19 as both a pandemic and public health emergency of international concern.

During the outbreak of Ebola and severe acute respiratory syndrome (SARS), digital health (DH) demonstrated its potential in detecting and fighting global epidemics [2,3,4]. DH is defined as technology that, “connects and empowers people and populations to manage health and wellness, augmented by accessible and supportive provider teams working within flexible, integrated, interoperable, and digitally-enabled care environments that strategically leverage digital tools, technologies, and services to transform care delivery” [5].

Recently, a significant number of DH efforts have emerged because of the unprecedented global strain of COVID-19 on healthcare systems. This article reveals that digital technologies can be used at different stages of the COVID-19 outbreak, including data-driven disease surveillance, screening, triage, diagnosis, and monitoring. Methods that could potentially reduce the exposure of healthcare providers to the virus will also be discussed. The review aims to guide further development in DH to improve infectious disease control. 

### 1.1. Surveillance

The WHO defines public health surveillance as, “the continuous, systematic collection, analysis, and interpretation of health-related data needed for the planning, implementation, and evaluation of public health practice” [6]. Advancements in information technology and information sharing is giving rise to a new field known as infodemiology. Infodemiology is defined as, “the science of distribution and determinants of information in an electronic medium, specifically the Internet, with the ultimate aim to inform public health and public policy” [7]. The Allen Institute for Artificial Intelligence (AI) is currently offering free access to a collection of machine-readable literature that includes information regarding the group of coronaviruses [8]. The Allen Institute database currently contains more than 44,000 articles, of which 29,000 are full-text publications. Combining this database with other unstructured data from websites or social media can be used to expand our knowledge further regarding the early detection and containment of future outbreaks of COVID-19 and other emerging infectious diseases. 

Online surveillance-mapping tools, such as the Surveillance and Outbreak Response Management and Analysis System (SORMAS) [9], as well as HealthMap [10], have the potential to improve the early detection of infectious diseases in comparison to traditional epidemiological tools [2]. SORMAS and HealthMap are currently being used for the surveillance of COVID-19. Similarly, BlueDot’s outbreak risk software, a modern epidemiological tool, was reported as the first organization to reveal news of the outbreak [11].

Both medical devices and wearables have the potential to be repurposed to detect emerging patterns that are indicative of disease outbreaks. For example, Fitbit devices have been used to inform timely and accurate models of population-level influenza trends [12]. Additionally, smart thermometers have provided a novel source of information for influenza surveillance and forecasting [13]. 

Colubri et al. [14] used machine learning to harmonize several data sets from the Ebola virus epidemic to provide informed access to evidence-based guidelines. These guidelines were then incorporated in the Ebola Care Guidelines app [14]. Many of these lessons and models can be re-applied to develop a similar app for COVID-19. The app has the potential to include real-time updates of evidence-based guidelines during a global pandemic to inform the general population and healthcare providers. The integration of real-time updates into electronic medical records can also act as a dependable resource for practicing healthcare providers. 

### 1.2. Screening

The current COVID-19 outbreak has spread internationally, which has prompted the WHO to demand the detection and management of suspected cases at points of entry into a country [15]. Screening travelers based on flight or cruise origin and travel history for cases of COVID-19 could allow individuals to receive the necessary treatment sooner and may limit the spread of the disease. 

The use of technology has the potential to screen travelers based on symptoms and travel history. For example, Taiwan integrated its national health insurance database with its immigration and customs database to create a “big data” resource for analytics [16]. The database was used to classify travelers’ infectious risks and it generated real-time alerts during clinical visits to aid in case identification [16]. It can also be utilized to test patients for COVID-19 who had previously tested negative for influenza; one COVID-19 case was confirmed from 113 patients who had already undergone influenza screening [16].

During the SARS pandemic, many countries instituted border measures to control the outbreak [17]. Several researchers stated that thermal image scanning has the potential to enhance screening for infectious diseases [17,18,19]. A mass temperature screening solution utilizing AI was developed to reduce the need for manual temperature screening. Integrated Health Information Systems and KroniKare are currently piloting a screening solution that automatically screens and identifies patients with symptoms such as fever [20]. Sun et al. [21] recommend using a microwave radar to capture heart rate and respiratory rate to enhance the accuracy of mass screening. Additional research is needed to validate and develop mass temperature screening solutions. 

### 1.3. Triage

In response to the viral outbreak, many countries have transitioned to virtual medical care. Online tools are used to prioritize the treatment of patients based on the severity of their condition. Digital stethoscopes, such as those from TytoCare [22] and Eko [23], could enhance the quality of remote medical exams. Such DH interventions aim to prove early access to healthcare and reduce the risk of transmission to other patients and healthcare providers in the hospital setting. 

Many countries and health institutions are offering free triage telehealth assessments for COVID-19. The telehealth assessments provide patients with access to websites or mobile apps, which consist of a short survey regarding the patient’s current condition. The survey includes questions about age, symptoms, and travel history. Based on their results, the respondents will be provided with tips, asked to visit a nearby mobile COVID-19 testing site or hospital, or become connected digitally with a healthcare provider. Furthermore, health-focused chatbots, such as Buoy Health [24] and Lark Health [25], can also help individuals interpret their symptoms and suggest appropriate next steps. Survey results could potentially be integrated with electronic medical records to assure continuity of care.

### 1.4. Diagnosis

Following digital triage, patients could benefit from an at-home diagnostic service. Similar to over-the-counter genetic or urinary tract infection tests, patients could receive a test kit via mail. The use of a drone delivery method could also be considered as a potential and efficient solution to ensure both a timely diagnosis and patient confidentiality. Some test kits may require laboratory analysis [26,27], while other tests could utilize a smartphone to assist in the interpretation of test results [28]. If results are positive, patients could receive a virtual consultation to explain the next steps. Additionally, all positive results should be communicated and reported to the appropriate agency. At-home diagnostic test kits seem promising and have the potential to reduce the pressure on the healthcare system. However, the United States Food and Drug Administration stated that as of 20 March 2020, there had been no authorization for the use of an at-home diagnostic tool for COVID-19 [29].

Computed tomography (CT) images are currently being used to confirm cases of COVID-19 [30]. Researchers were able to develop a deep learning model that can accurately detect COVID-19 and differentiate it from community-acquired pneumonia and other forms of lung disease [31]. The deep learning model can also be used remotely by medical professionals outside of the epidemic areas.

Furthermore, significant advancements in DH, specifically in the field of personalized medicine, have evolved in recent years. Researchers are currently working on developing rapid diagnostic tools, vaccines, and medications to cure and limit transmission of COVID-19 [32]. 

### 1.5. Monitoring

A patient’s measurements can be directly transmitted to healthcare providers or other monitoring entities through the use of remote monitoring technology. Remote monitoring technologies can connect wirelessly to networks via Bluetooth, WiFi, or cellular connection. An extensive body of literature exists regarding the use of DH in the remote monitoring of chronic diseases [33,34]. The same concept could also be applied to the monitoring of infectious diseases. Remote monitoring can be used to monitor individuals exposed to COVID-19 as well as close contacts of the individual. Remote monitoring can also be used to monitor the exposure of healthcare providers and high-risk patient populations. 

In essence, similar technology infrastructure currently used by remote monitoring programs can also be used to incorporate a thermometer for monitoring patients that are suspected of having COVID-19. The Sense Followup Ebola app has an automatic alert system for temperature readings ≥38 °C for individuals receiving follow-up care [35]. A similar feature could also be incorporated in future COVID-19 apps. Furthermore, the app could pair with advanced thermometers that allow continuous and real-time monitoring of changes in body temperature [36,37]. 

Other DH interventions have the potential to provide unique data to help us understand the possible effects of COVID-19 on patients with comorbidities. Steinhubl et al. [38] studied the use of a wearable, wireless “Band-Aid” sensor to monitor patients exposed to the Ebola virus. Additionally, Hexoskin repurposed biometric shirts that are capable of continuously measuring vital signs, including temperature, respiration effort, and cardiac activity, to better understand the evolution of COVID-19 and its effects on lung function [39]. The use of data from glucometers among patients with diabetes could also be used as an objective indicator of infection, as high glucose levels correlate with signs and symptoms of infection [40]. Infectious respiratory diseases, such as COVID-19, have the potential to worsen symptoms of pre-existing lung disease, and thus exacerbate the use of emergency medications. Increased use of inhaled emergency medication can be detected using a smart inhaler [41]. 

### 1.6. Contact Tracing

As researchers continue to work on vaccines and methods of treatment for COVID-19, the primary measure of containment is the interruption of human-to-human transmission. Contact tracing is the process of identification and follow-up of individuals who have been in contact with a person confirmed to have been infected with COVID-19. Traditional paper-based methods of contact tracing during the Ebola outbreak have been proven to be insufficient. Such methods caused incomplete identification of contacts and delays in contact tracing steps, such as the identification of contacts involved in suspected cases that required isolation [42]. 

Our smartphones are increasingly unlocking the power of AI and machine learning to provide accurate and real-time insight into various aspects of healthcare. Smartphones can utilize GPS or Bluetooth technologies for contact tracing purposes. Although contact tracing may seem challenging, previous epidemics have been effectively controlled through contact tracing and isolation initiatives [3,42]. To assist in COVID-19 contact tracing, a mobile app called TraceTogether is currently being used in Singapore [43]. The app uses Bluetooth technology to identify individuals that have been in close contact with patients who have been diagnosed with COVID-19.

## 2. Considerations for Healthcare Providers

Healthcare providers are considered a high-risk group for contracting COVID-19 [44]. The aim of DH implementation during a global pandemic is to reduce the risk of transmission to healthcare providers. The technology applications mentioned above have the potential to reduce transmission by minimizing face-to-face contact between clinicians and patients. Furthermore, DH interventions enable healthcare providers to fight the global pandemic, even if they are practicing self-isolation measures or working remotely. Both Aiva [45] and Deloitte assistant [46] have created an AI-enabled patient communication solution that allows patients to request assistance, via Amazon Alexa or Google Home, without the need to press the traditional call bell button. Such interventions have the potential to reduce transmission because healthcare providers may be able to respond to many of the patient’s needs without having to enter the patient’s room. Thus, potentially reducing the frequency of a healthcare provider’s exposure. 

## 3. Challenges

It is important to note that the DH implementation process is likely to be challenging and resource-intensive. For example, the lack of homogenous interventions poses a significant challenge in applying DH interventions. The lack of homogeneity leads to variations in the nature of DH interventions and data collection methods. Such differences could limit model generalizability and limit understandings of the effectiveness of DH. Governments will need to work with all experts and stakeholders to embed secure DH interventions into practice while maintaining the privacy and confidentiality of patients. It is also important to consider that some countries may not have the technological infrastructure to support DH. Furthermore, there will be a significant proportion of the population who will not have access to technology or internet connectivity. 

## 4. Implications for Practice

This commentary provides valuable insights regarding various DH interventions that can be implemented to enhance the detection and spread of infectious diseases, such as COVID-19. This information may help a variety of stakeholders—including epidemiologists, healthcare professionals, and policymakers—who are planning to use DH to tackle infectious diseases. The majority of DH interventions have already been developed for other infectious diseases, such as Ebola, SARS, and the flu. However, many governments and health systems have been slow in adopting these technologies.

Government, professional associations, and health organizations should take an active role in DH adoption. The United Nations established the Panel on Digital Cooperation to address challenges in the digital age and propose modalities for working cooperatively across sectors, disciplines, and borders, to address challenges in the digital age [47]. This panel could work closely with the WHO to help address the challenges of implementing digital health in the context of infectious diseases. These organizations could help in addressing the digital divide among countries with limited technological infrastructure. This can be accomplished by sharing the research and development protocols and source codes, similar to SROMAS [9] and Colubri et al. [14].

Additional research is required to further assess the effectiveness of DH in detecting and managing arising infectious diseases. In addition, cost-effectiveness analysis is required to assess the impact of DH on healthcare resources.

## 5. Conclusions

Digital Health provides an opportunity to use real-time data to improve the prevention and control of the rapidly changing nature of epidemics. Recent SARS, H1N1, and Ebola outbreaks offer many lessons about the use of DH for public health emergencies. These learnings can be transferred to new effective technologies to enhance our response against the COVID-19 pandemic. DH has the potential to strengthen our preparedness for the next pandemic. We need to have these tools locked and loaded for our next war against infectious disease.

## References

[1] Zhu N., Zhang D., Wang W., Li X., Yang B., Song J., Zhao X., Huang B., Shi W., Lu R. (2020). A Novel Coronavirus from Patients with Pneumonia in China, 2019. N. Engl. J. Med..

[2] Bempong N.E., De Castañeda R.R., Schütte S., Bolon I., Keiser O., Escher G., Flahault A. (2019). Precision Global Health—The case of Ebola: A scoping review. J. Glob. Health.

[3] Tom-Aba D., Nguku P., Arinze C., Krause G. (2018). Assessing the Concepts and Designs of 58 Mobile Apps for the Management of the 2014-2015 West Africa Ebola Outbreak: Systematic Review. JMIR Public Health Surveill..

[4] Eysenbach G. (2003). SARS and Population Health Technology. J. Med. Internet Res..

[5] HIMSS Defines Digital Health for the Global Healthcare Industry Healthcare Information and Management Systems Society. https://www.himss.org/news/himss-defines-digital-health-global-healthcare-industry.

[6] Public Health Surveillance World Health Organization. https://www.who.int/topics/public_health_surveillance/en/.

[7] Eysenbach G. (2011). Infodemiology and Infoveillance. Am. J. Prev. Med..

[8] COVID-19 Open Research Dataset (CORD-19) Semantic Scholar. https://pages.semanticscholar.org/coronavirus-research.

[9] SORMAS the Surveillance, Outbreak Response Management and Analysis System Published 2020. https://sormasorg.helmholtz-hzi.de/.

[10] Contagious Disease Surveillance Healthmap. https://healthmap.org/en/.

[11] Bogoch I., Watts A., Thomas-Bachli A., Huber C., Kraemer M., Khan K. (2020). Potential for global spread of a novel coronavirus from China. J. Travel Med..

[12] Radin J., Wineinger N., Topol E., Steinhubl S. (2020). Harnessing wearable device data to improve state-level real-time surveillance of influenza-like illness in the USA: A population-based study. Lancet Digital Health.

[13] Miller A., Singh I., Koehler E., Polgreen P. (2018). A Smartphone-Driven Thermometer Application for Real-time Population- and Individual-Level Influenza Surveillance. Clin. Infect. Dis..

[14] Colubri A., Hartley M., Siakor M., Wolfman V., Felix A., Sesay T., Shaffer J.G., Garry R.F., Grant D.S., Levine A.C. (2019). Machine-learning Prognostic Models from the 2014–16 Ebola Outbreak: Data-harmonization Challenges, Validation Strategies, and mHealth Applications. EClinicalMedicine.

[15] Management of Ill Travellers at Points of Entry ( International Airports, Seaports, and Ground Crossings) in the Context of COVID-19, Interim Guidance, 19 March 2020 Published 2020. Apps.who.int..

[16] Wang C., Ng C., Brook R. (2020). Response to COVID-19 in Taiwan. JAMA.

[17] Selvey L., Antão C., Hall R. (2015). Evaluation of Border Entry Screening for Infectious Diseases in Humans. Emerg. Infect Dis..

[18] Cho K., Yoon J. (2014). Fever Screening and Detection of Febrile Arrivals at an International Airport in Korea: Association among Self-reported Fever, Infrared Thermal Camera Scanning, and Tympanic Temperature. Epidemiol. Health.

[19] Priest P., Duncan A., Jennings L., Baker M. (2011). Thermal Image Scanning for Influenza Border Screening: Results of an Airport Screening Study. PLoS ONE.

[20] iThermo KroniKare. https://kronikare.ai/ithermo/.

[21] Sun G., Nakayama Y., Dagdanpurev S., Abe S., Nishimura H., Kirimoto T., Matsui T. (2017). Remote sensing of multiple vital signs using a CMOS camera-equipped infrared thermography system and its clinical application in rapidly screening patients with suspected infectious diseases. Int. J. Infect. Dis..

[22] On Demand Medical Exams Anytime. Anywhere. TytoCare. https://www.tytocare.com/.

[23] Noise Canceling AI-Powered Stethoscopes + ECGs Ekohealth.com. https://www.ekohealth.com/.

[24] Symptom Checker, Check Your Symptoms in Real Time Buoyhealth.com. https://www.buoyhealth.com/symptom-checker/.

[25] Digital Disease Management & Prevention Platform Lark Health. https://lark.com/.

[26] Innovative at-home Health Testing Everlywell.com. https://www.everlywell.com/..

[27] Modern Primary & Urgent Care Carbon Health. https://carbonhealth.com/..

[28] At Home Medical Testing Kits Scanwellhealth. https://www.scanwellhealth.com/.

[29] Coronavirus (COVID-19) Update: FDA Alerts Consumers About Unauthorized Fraudulent COVID-19 Test Kits Published 2020. https://www.fda.gov/news-events/press-announcements/coronavirus-covid-19-update-fda-alerts-consumers-about-unauthorized-fraudulent-covid-19-test-kits.

[30] Bernheim A., Mei X., Huang M., Yang Y., Fayad Z.A., Zhang N., Diao K., Lin B., Zhu X., Li K. (2020). Chest CT Findings in Coronavirus Disease-19 (COVID-19): Relationship to Duration of Infection. Radiology.

[31] Li L., Qin L., Xu Z., Yin Y., Wang X., Kong B., Bai J., Lu Y., Fang Z., Song Q. (2020). Artificial Intelligence Distinguishes COVID-19 from Community Acquired Pneumonia on Chest CT. Radiology.

[32] Pang J., Wang M., Ang I., Tan S.H.X., Lewis R.F., Chen J.I.-P., Gutierrez R.A., Gwee S.X.W., Chua P.E.Y., Yang Q. (2020). Potential Rapid Diagnostics, Vaccine and Therapeutics for 2019 Novel Coronavirus (2019-nCoV): A Systematic Review. J. Clin. Med..

[33] Alwashmi M., Hawboldt J., Davis E., Marra C., Gamble J., Abu Ashour W. (2016). The Effect of Smartphone Interventions on Patients with Chronic Obstructive Pulmonary Disease Exacerbations: A Systematic Review and Meta-Analysis. JMIR Mhealth Uhealth.

[34] Noah B., Keller M., Mosadeghi S., Stein L., Johl S., Delshad S., Tashjian V.C., Lew D., Kwan J.T., Jusufagic A. (2018). Impact of remote patient monitoring on clinical outcomes: An updated meta-analysis of randomized controlled trials. NPJ Digit. Med..

[35] Sense Followup Ehealthafrica. http://ehealthafrica.github.io/case-studies/sense-followup.html.

[36] TempTraq Vermed.com. https://vermed.com/MedicalDevices/TempTraq.aspx.

[37] Fever Scout Vivalnk.com. http://www.vivalnk.com/feverscout.

[38] Steinhubl S., Marriott M., Wegerich S. (2015). Remote Sensing of Vital Signs: A Wearable, Wireless “Band-Aid” Sensor with Personalized Analytics for Improved Ebola Patient Care and Worker Safety. Glob. Health Sci. Pract..

[39] Hexoskin Smart Shirts—Cardiac, Respiratory, Sleep & Activity Metrics Hexoskin. https://www.hexoskin.com/.

[40] Berbudi A., Rahmadika N., Cahyadi A., Ruslami R. (2019). Type 2 Diabetes and its Impact on the Immune System. Curr. Diabetes Rev..

[41] Breathesuite Published 2020. https://www.breathesuite.com/.

[42] Danquah L., Hasham N., MacFarlane M., Conteh F.E., Momoh F., Tedesco A.A., Jambai A., Ross D.A., Helen A. (2019). Weiss. Use of a mobile application for Ebola contact tracing and monitoring in northern Sierra Leone: A proof-of-concept study. BMC Infect. Dis..

[43] TraceTogether—Behind the Scenes Look at Its Development Process GOVTECH Singapore. https://www.tech.gov.sg/media/technews/tracetogether-behind-the-scenes-look-at-its-development-process.

[44] Wang C., Horby P., Hayden F., Gao G. (2020). A novel coronavirus outbreak of global health concern. Lancet.

[45] Virtual Health Assistant Aiva Health. https://aivahealth.com/.

[46] DeloitteASSIST Deloitte New Zealand. https://www2.deloitte.com/nz/en/pages/life-sciences-and-healthcare/articles/deloitte-assist.html.

[47] The High-Level Panel on Digital Cooperation Published 2020. https://digitalcooperation.org/.

